# Euthymia scale as a protective factor for depressive symptoms: a one-year follow-up longitudinal study

**DOI:** 10.1186/s13104-023-06512-x

**Published:** 2023-09-22

**Authors:** Natsu Sasaki, Daisuke Nishi

**Affiliations:** Department of Mental Health, Graduate School of Medicine, The University of Tokyo, 7-3-1 Hongo, Bunkyo-Ku, Tokyo, 1130033 Japan

**Keywords:** Well-being, Depression, Prevention, Recurrence, Psychiatry

## Abstract

**Objectives:**

This study aimed to examine whether a high score on the euthymia scale (ES) predicts a low incidence of depressive symptoms one year later.

**Methods:**

The baseline online survey was conducted in February 2020, and a follow-up survey was done in February 2021. Japanese over 20 years old were enrolled. Respondents who answered both baseline and follow-up, and without depressive symptoms at baseline were included in the analysis. The euthymia scores at baseline was measured by the 10 items of the Japanese version of the ES. Depressive symptoms at follow-up were determined if participants showed either depressive feelings or anhedonia. The odds ratio (OR) was calculated using multivariate logistic regression analysis, adjusting for age, gender, marital status, educational attainment, and clinical visit for depressive episode before the baseline survey.

**Results:**

The total of 624 participants were analyzed. A total of n = 63 (10.1%) presented depressive symptom at follow-up. A high ES score significantly predicted a lower incidence of depressive symptoms, after adjusting for covariates (aOR = 0.81 [95% confidence interval: 0.72–0.89]). Using the cutoff score retrieved from this data, a high ES score (7 or more) showed the same tendency, compared to a low ES score (< 7) (aOR = 0.46 [0.25–0.83]).

**Conclusions:**

This study suggests the predictive usefulness of euthymia for subsequent depressive symptoms. Further investigation is needed by employing rigid diagnostic criteria.

## Introduction

Depression is a major public health concern and is a growing cause of disability, social burden, and negative health outcomes. Depressive symptoms also further increase the risk of subsequent cardiovascular diseases [1], dementia [2], and cancer mortality [3]. Moreover, subclinical depression is more highly prevalent than depression [4]. A condition of having depressive symptoms that do not meet the criteria for a depressive disorder is associated with health problems [5], decreased quality of life [6], high economic cost [7], and precedented major depression [8, 9]. Thus, successful prevention is important at early stages of depression. Detecting a condition of having depressive symptoms in a few items are of benefit in practical use [10, 11]. Moreover, modifiable factors that can be easily assessed in clinical practice should be targeted to prevent depression [12]. However, indicators of vulnerability which should be targeted for effective preventive intervention are not adequately available [13].

Euthymia is a newly developed concept that has garnered high attention as a clinimetric. Euthymia is a trans-diagnostic construct characterized by a lack of mood disturbances; presence of positive affect; balance of psychological well-being dimensions, flexibility, consistency, and resistance to stress [13]. Euthymia does not consist of only one component, but rather is an integrative and unifying framework that considers psychopathological interactions [14], and clinical global judgement [15]. The Euthymia scale (ES) is a comprehensive clinimetric instrument for assessing euthymia that is comprised of ten items [16, 17]. Cross-sectionally, ES showed negative associations with depressive symptoms [18] and psychological distress [19]. Moreover, ES is sensitive to discrimination between subclinical depressive symptoms and a major depressive episode [19], suggesting the possible ability to become a comprehensive assessment of recovery. Previous studies suggest that euthymia decreases vulnerability to future deterioration [14, 20]. Considering its unifying concepts, euthymia may play an important role in prevention for newly developed depressive symptoms. However, there are currently no studies prospectively investigating whether euthymia scores show protectivity for future onset of depressive symptoms.

The aim of this study thus is to investigate whether a high ES score predicts a lower incidence of having depressive symptoms one-year later. In addition, we discuss an appropriate cut-off value for effective screening and as a target value for interventions.

## Methods

### Study design

This study utilized a longitudinal design and collected follow-up data one year after the baseline survey. The baseline survey was conducted online in February 2020 and a follow-up survey was conducted one year after the baseline in February 2021. The study was reviewed and approved by the Research Ethics Committee of Graduate School of Medicine/Faculty of Medicine, The University of Tokyo (no. 2019361NI-(3)).

### Participants

The participants of baseline survey were recruited from a registered panel of an online survey company, Macromill, Inc. (https://www.macromill.com/). From about 2.3 million potential participants representing all prefectures in Japan, 1030 respondents among eligible participants were included as baseline data in order of arrival to the form. Eligibility criteria were: (a) living in Japan and (b) 20 years of age or older. The aim of the initial survey was to compare euthymia scores between people with history of depression, but without current symptoms, and people without history nor current symptoms. Participants at baseline were sampled from two strata equally (about 50% vs. 50%) according to their history of major depressive episodes in their past life. The online research company sent an invitation email for panel members who registered as aged over 20 and living in Japan. The response form was closed when the target number of answers was reached. Informed consent was obtained from all participants via instructions on the survey and indicated disagreement by not answering the questionnaire. All participants (N = 1030) were invited to the follow-up survey one year after the baseline. The baseline survey was conducted in February 2020, and the follow-up survey was in February 2021. Participating monitors were awarded approximately 100 tokens (equivalent to 100 Japanese yen) as a reward in each survey.

The participants of this longitudinal study were included according to the following criteria: (a) answered both baseline and follow-up, and (b) without depressive symptoms at baseline.

### Measurement scales

#### Euthymia scale (ES)

The original English version of the ES-J rating scale is a 10 item self-reported questionnaire [13]. Each item of the ES-J is scored dichotomously as False (0) or True (1), resulting in an overall summed score ranging from 0 to 10, with higher scores indicating a better euthymic state. The Japanese version of the ES was tested for its reliability and validity and was published elsewhere [19].

#### Depressive symptoms

Depressive symptoms were assessed at baseline and follow-up by using two items for the past two weeks (“Were you depressed or down, or felt sad, empty or hopeless most of the day, nearly every day?” “Were you much less interested in most things or much less able to enjoy the things you used to enjoy most of the time?”), referring Whooley questions (a two-question instrument). The response options were No (0) or Yes (1). If the respondents answered yes for either item, we judged the respondents as having any of depressive symptoms. While Whooley questions do not cover all the variety of depressive symptoms and do not meet the criteria of the Diagnostic and Statistical Manual of Mental Disorders (DSM-5) for depression, it can be used as a practical tool for detecting conditions suspicious of depression [11].

#### Demographic variables

A questionnaire was administered to assess the following demographic variables: gender (male or female), age, marital status (married or single), education status (less than high school, college/vocational, undergraduate or over), and depressive symptoms before baseline survey. Depressive symptoms before baseline were assessed at the baseline survey as a history of major depressive episodes in the past life by using two items (depressed mood and loss of interest or pleasure).

### Statistical analysis

The group difference of the mean ES score at baseline was stratified by each demographic characteristic and was examined by using univariate analysis of variance.

In receiver–operator characteristic (ROC) curve analyses, sensitivity, specificity, positive and negative predictive values, and area under the curve (AUC) were calculated. An AUC of 0.9–1 is considered excellent, 0.8–0.9 good, 0.7–0.8 fair, 0.6–0.7 poor, and 0.5–0.6 similar to chance [21]. The ES cut-off value was determined using ROC curve analysis.

The odds ratio (OR) was calculated to examine the association between euthymia score at baseline and depressive symptom at follow-up by using multivariate logistic regression analysis, adjusting for age, gender, marital status, educational attainment, and clinical visit for depressive episode before baseline survey. Model 1 (continuous ES), Model 2 (using cutoff score), and Model 3 (four quantile) were examined and presented to show the odds of depressive symptoms.

Statistical significance was defined as p < 0.05. All the statistical analyses were performed using SPSS 28.0, Japanese version (IBM Inc., Chicago, IL).

## Results

The total of 624 participants were included in the study. Table 1 shows the participants’ characteristics. The mean age was 50.0 (standard deviation; 13.9). 43.1% of the participants reported that they had experienced a depressive episode in the past before baseline survey. The mean euthymia scale score stratified by basic demographic characteristics is shown in Table 2. A high ES was observed in people who were older (over 60 years old), married, and without history of a depressive episode.

**Table 1:** Participants’ Characteristics at Baseline (N = 624)

	N (%)	Mean (SD) [min—max]
Age		50.0 (13.9) [20–88]
20–29 years old	33 (5.3)	
30–39 years old	111 (17.8)	
40–49 years old	164 (26.3)	
50–59 years old	176 (28.2)	
Over 60 years old	140 (22.4)	
Gender		
Male	323 (51.8)	
Female	301 (48.2)	
Marital status		
Single	212 (34.0)	
Married	412 (66.0)	
Educational attainment		
Less than high school^(a)^	206 (33.0)	
College/Vocational	148 (23.7)	
Undergraduate or over	270 (43.3)	
Having depressive symptoms before baseline survey^(b)^		
Yes	269 (43.1)	
No	355 (56.9)	

*SD* standard deviation

(a) Respondents who answer “other” (n = 1) were included in less than high school

(b) Depressive symptoms before baseline survey were assessed by two items (depressed mood and loss of interest or pleasure) over two weeks in life so far. If the respondents answered yes for either item, we judged the respondents as having depressive symptoms

**Table 2:** Mean score of Euthymia Scale Stratified by Basic Demographic Characteristics (N = 624)

	Mean score of Euthymia scale at baseline [possible range 0–10]	Group difference
	Mean (SD)	p-value ^(a)^
Age		
20–29 years old	6.2 (3.1)	< 0.001*
30–39 years old	6.1 (2.8)	
40–49 years old	6.3 (3.0)	
50–59 years old	6.6 (2.8)	
Over 60 years old	7.9 (2.3)	
Gender		
Male	6.8 (2.9)	0.616
Female	6.6 (2.7)	
Marital status		
Single	6.3 (3.0)	0.008*
Married	6.9 (2.7)	
Educational attainment		
Less than high school^(b)^	6.5 (2.9)	0.576
College/Vocational	6.7 (2.6)	
Undergraduate or over	6.8 (2.9)	
Having depressive symptoms before baseline survey^(c)^		
Yes	5.6 (2.9)	< 0.001*
No	7.5 (2.5)	

*SD* standard deviation

^*^p < 0.05

(a) The group difference was examined by using univariate analysis of variance

(b) Respondents who answer “other” (n = 1) were included in less than high school

(c) Depressive symptoms before baseline survey were assessed by two items (depressed mood and loss of interest or pleasure) over two weeks in life so far. If the respondents answered yes for either item, we judged the respondents as having depressive symptoms

**Fig. 1:**
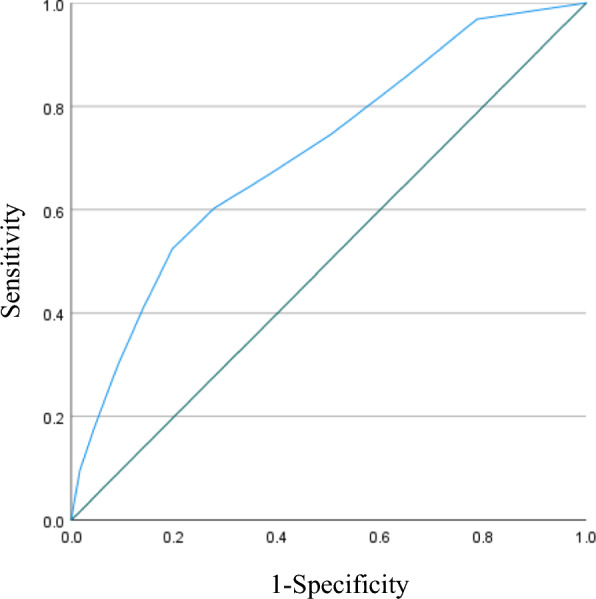
Receiver operating characteristic curve of euthymia score to predict depressive symptoms one year later

A total of n = 63 (10.1%) presented depressive symptoms at follow-up. Figure 1 shows the ROC curve of the euthymia score to predict depressive symptoms one year later. The AUC is 0.710 [95% confidential intervals: 0.642–0.777, p < 0.001], and the optimum predictive cut-off point of ES was a score of 6.5. Sensitivity, specificity, positive and negative predictive values were 67%, 62%, 16%, and 94%, respectively (Table 3). Since the ES score ranged 0 to 10 as an integer value, authors set the cut-off score of the ES on 7.

**Table 3:** Performance of Euthymia Score in Predicting Depressive Symptoms at 1-year Follow-up (N = 624)

Euthymia	Depressive symptoms at follow-up survey (1 year)	
	Negative	Positive
Low (< 7), n (%)	214 (83.6)	42 (16.4)
High (7 or more), n (%)	347 (94.3)	21 (5.7)
Value (95% CI)		
Sensitivity	0.67 (0.58–0.76)	–
Specificity	0.62 (0.52–0.72)	–
Positive predictive value	0.16 (0.09–0.23)	–
Negative predictive value	0.94 (0.89–0.99)	–

*CI* confidence intervals

The association of ES at baseline with depressive symptoms at follow-up is presented in Table 4. In model 1 (continuous), a high ES score significantly predicted a lower incidence of depressive symptoms, after adjusting covariates (OR = 0.81 [95% confidence interval: 0.72–0.89]). In model 2, using a cutoff score of 7, a high ES score (7 or more) showed the same tendency (OR = 0.46 [0.25–0.83]). In model 3, using the quantile, the highest score of a quantile (ES score 9 or 10) showed the lowest odds (OR = 0.24 [0.11–0.55]), compared to the first quantile (score 0–3).

**Table 4:** Odds ratio for Having Depressive Symptoms at Follow-up Survey by Multivariate Logistic Regression Analysis (N = 624)

	N	Crude		Adjusted^(a)^	
		OR	95% CI	OR	95% CI
Model 1(continuous)					
Euthymia score	624	0.77	0.70–0.84	0.81	0.72–0.89
Model 2 (cut-off)^(b)^					
Low (score < 7)	256	1.00		1.00	
High (7 or more)	368	0.31	0.18–0.54	0.46	0.25–0.83
Model 3 (quantile)^(c)^					
Quantile 1 (score 0–3)	105	1.00		1.00	
Quantile 2 (4–6)	151	0.36	0.18–0.71	0.38	0.19–0.77
Quantile 3 (7–8)	150	0.24	0.11–0.51	0.32	0.15–0.72
Quantile 4 (9, 10)	218	0.15	0.07–0.32	0.24	0.11–0.55

*OR* odds ratio, *CI* confidence interval

(a) Adjusted for age, gender, marital status, educational attainment, and having depressive symptoms before baseline survey

(b) Euthymia score was dichotomized by using the median

(c) Euthymia score was divided into four categories by using the quantile

## Discussion

This study investigated the utility of ES as a predictor of subsequent incidence of depressive symptoms one year later. The findings showed that a high ES could predict a low risk of future depressive symptoms. Sensitivity, specificity and AUC of ES were fair and acceptable. The brief items for ES are an advantage for practical use.

This study found that high ES was observed in persons who were older, married, and without history of a depressive episode. These are well-known protective factors for mental health. ES measures the optimal balance of well-being according to changing needs, including resilience and flexibility [14]. The present findings are partially in line with previous studies reporting that resilience is high in the older population [22, 23], and people with high social support [24]. In addition, it is already known that ES is high in people without a history of depression [19]. Focusing on modifiable factors, rather than unmodifiable basic characteristics or events in early life, can be applied to prevent depression among individuals at elevated risk [25]. ES can be a proximal indicator to assess protective factors appropriately, without using unmodifiable variables.

The current study examined the utility of the ES scale for identifying a condition of not having depressive symptoms one year later. ROC curve analysis identified an ES sore 7 as optimal in screening respondents without a future risk of having depressive symptoms. Using the identified cut-point of the ES score of 7, the negative predictive value was high (94%), suggesting that people with the ES score of 7 or more have a very high likelihood of not experiencing any of the two depressive symptoms (depressed mood and loss of interest or pleasure) within a year. The odds ratio of a high ES score (7 or more) for an incidence of depressive symptoms was 0.46, compared to a low score (< 7). That of highest score of the ES (9 or 10) was 0.24, compared to scores of 0–3. The whole population in this study had no depressive symptoms at baseline. Such a difference in the ES score at baseline may reflect potential resilience, which needs to be a point of focus in the intervention. We should note that the positive predictive value for those with low score (< 7) was only 16.4%, suggesting that the ability of ES-J as a screening tool to detect a condition of having depressive symptoms is low.

## Practical implications

The present study showed that those with high ES-J scores were less likely to have a depressive symptom one year later, regardless of the history of depression. In this sense, euthymia can apply to preventive interventions in community, workplace, and perinatal care settings, including relapse prevention at clinical settings. The pursuit of euthymia can be achieved by psychotherapeutic techniques aiming to enhance positive affects and psychological well-being (such as well-being therapy, mindfulness-based cognitive therapy, and acceptance and commitment therapy) [26]. The ES-J scores may be used as an indicator of the effectiveness of preventive interventions.

## Limitations

This study has several limitations. First, the sample size was not determined in *priori*. Given the AUC (0.710) with 80% power and 95% confidence and a likelihood ratio of 9, the total number of participants needed was estimated n = 259. Second, generalizability was also limited due to being an online survey; people with high IT literacy and device ownership were more likely to be included. People who registered in an online survey company may have a motivation to spend time to answer questionnaires and get small amount of money, suggesting the biased population. The participants were not representative of the Japanese population. Third, outcome of depressive symptoms was assessed by only two items (depressed mood and loss of interest or pleasure) based on Whooley questions. The simplified judgment criteria might overestimate the depressive symptoms in our study. The two-question instrument has been reported as a sensitivity of 96%, a specificity of 57%, and a positive predictive value of 33%, making it a test with a relatively high false-positive rate. In this regard, serious concerns exist about using the two-question instrument as the golden standard for determining depression. Besides, we did not exclude other conditions that can present with depressive symptoms, such as bipolar disorder and schizophrenia. Future studies should apply clinically robust methods to determine the outcomes. The severity of the symptoms was also not assessed in the study, leading to possible overestimation of a condition of having depressive symptoms. Fourth, the proportion of participants who experienced a depressive episode before the baseline survey of this study was higher than general population, since the half of the participants at baseline was recruited from those with a history of major depressive episodes. The generalizability of the present findings is limited regarding the heterogeneity of the study population. Sampling bias may also cause an overestimation of the validity of ES-J.

## Conclusion

This study investigated whether a high ES score predicts a low incidence of subclinical depression one year later. Additionally, a cut-off point was determined for screening. A high ES score significantly predicted a lower incidence of depressive symptoms, after adjusting for covariates. The brief ten-items from ES are potentially useful for effectively assessing protective aspects for depression. Future study should examine whether the findings can be reproduced in different populations other than the Japanese. Those under highly disadvantaged uncontrolled environment may lead different findings.

## Data Availability

The data used in this study are not available in a public repository because they contain personally identifiable or potentially sensitive patient information. All data can be provided by DN, upon reasonable request.
